# Surface Modification of Organic Chromium-Free Tanned Leather Shavings and the Immobilization of Lipase

**DOI:** 10.3390/polym17050688

**Published:** 2025-03-04

**Authors:** Dongyan Hao, Xuechuan Wang, Jiajia Shi, Zhisheng Wang, Xing Zhu

**Affiliations:** 1 School of Chemical Engineering and Modern Materials, Shangluo University, Shangluo 726000, China; 19929931828@163.com; 2 College of Bioresources Chemical and Material Engineering, Shaanxi University of Science and Technology, Xi′an 710021, China; wangxc@sust.edu.cn (X.W.); 13891137017@163.com (J.S.)

**Keywords:** leather scraps, enzyme immobilization, lipase hydrolysis, waste grease

## Abstract

Following the concept of “waste into resources”, a mild and controllable light grafting technique was used to immobilize pancreatic lipase (PPL) in situ on modified organic, chromium-free tanned leather scraps to catalyze the hydrolysis of waste oil. The experimental results showed that immobilized PPL significantly improved the catalytic activity, operational stability, reusability, and storage stability compared to free PPL. Furthermore, the study evaluated the environmental compatibility of the system through biological risk assessment of soil extracts after degradation, indicating that the system has good environmental compatibility. The experiment is simple to operate, uses mild conditions, and the immobilized material is obtained from leather-making solid waste. The use of this immobilization system to treat waste oil in the leather-making process is of great significance for achieving clean and sustainable production in the leather industry.

## 1. Introduction

During leather manufacturing, processes such as degreasing and fleshing generate millions of tons of waste oil annually. Improper disposal of these lipid-rich byproducts not only triggers severe environmental contamination through soil degradation and water eutrophication but also represents a significant loss of valuable biomass resources—creating a paradox between industrial development and ecological sustainability [1,2,3]. Conventional treatment strategies, which are based on chemical catalysis under high-temperature/pressure conditions (200–300 °C, 5–10 MPa) face critical limitations, including energy-intensive operations (typically requiring 2.5–3.5 MJ/kg), catalyst deactivation issues, and secondary pollution from toxic byproducts like polycyclic aromatic hydrocarbons [4,5,6,7,8]. These technical bottlenecks fundamentally are inconsistent with the principles of green chemistry and circular economy [9,10].

Compared to chemical reagents, the hydrolysis of oil catalyzed by lipase is a safe, green, efficient, and clean biological treatment method [11,12,13]. Under the action of lipase, oil will be specifically hydrolyzed by the lipase system, and the products can be converted into biodiesel [14,15,16]. However, in the actual production process, the application of lipase hydrolysis of industrial waste oils has been greatly impeded due to the shortcomings of lipase, such as high cost, poor operational stability, and recyclability [17,18,19,20]. Enzyme immobilization is the restriction or fixation of an enzyme to a specific spatial area by physical or chemical methods, which provides a viable way to solve the problem of high cost and difficult recovery of free enzymes [21,22,23]. The immobilized enzymes can be separated from the final product very easily and conveniently, reducing contamination of the product [24,25].

**Figure 1:**
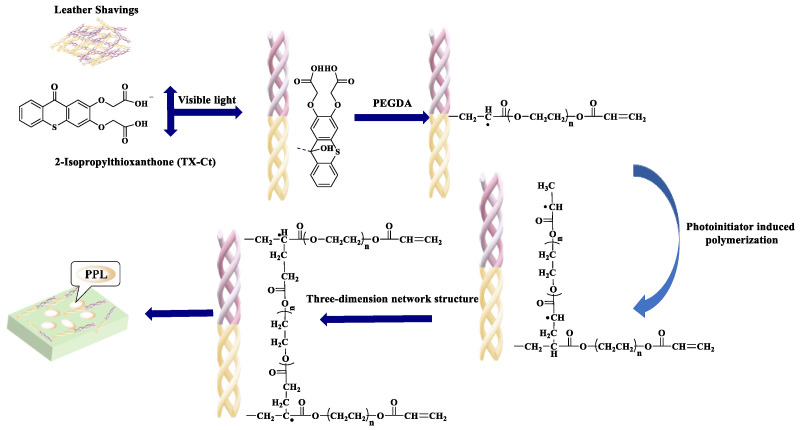
Schematic diagram of in situ lipase embedding by visible light graft polymerization.

In this context, a novel immobilization platform was developed utilizing chromium-free organic tanned leather shavings—a collagen-based waste material from tanning operations—as a sustainable matrix. Through visible light-induced graft polymerization, polyethylene glycol (PEG) hydrogels are covalently anchored onto the shavings’ surface, creating a hierarchical structure for lipase immobilization (Figure 1). This design capitalizes on three synergistic effects: (1) The microporous collagen network (10–50 μm pore size) provides a high surface area for enzyme loading, (2) PEG hydrogel’s thermoresponsive properties facilitate substrate diffusion, and (3) the biodegradable leather matrix ensures environmental compatibility [26,27,28,29]. Comprehensive characterization via SEM-EDS, XPS, and in situ FT-IR confirmed successful hydrogel grafting and enzyme fixation. Systematic evaluation of the hybrid biocatalyst demonstrated remarkable features: 82.4% relative activity retention after 15 batch cycles, 68% soil mineralization within 60 days, and 93.5% hydrolysis efficiency for waste oils compared to free enzymes. These findings not only establish a closed-loop strategy for concurrent waste oil and leather shavings valorization but also provide mechanistic insights into designing next-generation green biocatalytic systems for the leather industry.

## 2. Materials and Methods

### 2.1. Materials

Polyethylene glycol diacrylate (PEGDA molecular weight: 575) and dodecanoic acid, 4-nitrophenylester (AR) were supplied by Sigma Shanghai Co., Ltd. (Shanghai, China). Concentrated sulfuric acid and potassium chloride (AR) were purchased from Beijing Chemical Reagent Co., Ltd. (Beijing, China). Thiosalicylic acid (AR), potassium dihydrogen phosphate, and catechol-*O*,*O*-diacetic acid (AR) were supplied by Shanghai Alfa Esha Co., Ltd. (Shanghai, China). Disodium hydrogen phosphate (AR), P-Nitrophenol laurate (p-NPL, AR), and sodium hydroxide granules (AR) were bought from Shanghai Maclean Biochemical Technology Co., Ltd. (Shanghai, China). Coomassie Brilliant Blue Solution G-250(AR) was purchased by Isegu Biotechnology Co., Ltd. (Suzhou, China). Leather shavings were provided by Henan Longfeng Leather Co., Ltd. (Henan, China). Lipase from porcine pancreas (PPL) was purchased from Shanghai Huchen Industrial Co., Ltd. (Shanghai, China). Castor Oil (AR) was purchased from Shantou Xilong Science Co., Ltd. (Shantou, China).

### 2.2. Methods

#### 2.2.1. Synthesis of Photoinitiator TX-Ct

Concentrated sulfuric acid (26.70 mL) was added into a three-neck flask, cooled, and stirred in an ice bath for 35 min, and then 3.7 mmol thiosalicylic acid was dissolved in the solution. Then, 0.01 mol of catechol-*O*-*O*-peroxydiacetic acid was added in batches within 15 min while stirring, and the reaction was carried out under light protection for 73 h. After the reaction, the reaction solution was slowly added to 200 mL of ice water (0 °C) for 12 h to obtain TX-Ct solution, and then the yellow powder initiator TX-Ct was obtained after centrifugation and drying [30].

#### 2.2.2. Lipase Immobilization

A photoinitiator solution was prepared by dissolving 10 mg of TX-Ct initiator in 10 mL of PBS buffer (0.05 mol/L, pH 7.7). Subsequently, 3 mL of this solution was added to the reaction system, followed by 10 mg of leather chip powder as the carrier. A PPL solution (0.87 mg/mL) was prepared using PBS, and 1.5 mL of this solution, along with a 25% (*v*/*v*) PEGDA solution, was sequentially added to the flask. The immobilization of lipase was carried out under visible light. A schematic diagram of the in situ lipase embedding process via visible light-induced graft polymerization is shown in Figure 1.

### 2.3. Testing and Characterisation

#### 2.3.1. Scanning Electron Microscope (SEM)

SEM (Hitachi, Tokyo, Japan, Regulus 8100) was used to observe the microstructure of immobilized PPL and blank leather shavings.

#### 2.3.2. X-Ray Photoelectron Spectroscopy Test (XPS)

To confirm the successful encapsulation of PEGDA on the surface of leather chips, we performed XPS (Thermo Fisher, Waltham, MA, USA, NEXSA) on immobilized PPL and blank leather chips to obtain their C1s and N1s spectra.

#### 2.3.3. Fourier Transform Infrared Spectroscopy Test (FT-IR)

To characterize the leather chips with surface-encapsulated PEGDA and immobilized PPL, FT-IR (Bruker, Ettlingen, Germany, Vertex70) was performed on immobilized PPL and blank leather chips to obtain their FT-IR spectra.

#### 2.3.4. Ultra-Depth-of-Field Microscope Test

The surface morphology of blank and fixed PPL chips was observed by an ultra-depth-of-field 3D microscope (VHX-700F, Kiento Co., Ltd., Osaka, Japan).

### 2.4. Studies on the Catalytic Properties of Immobilized Lipases

The immobilized enzyme was placed in PBS buffer at pH 9, and then put into a centrifuge tube and stored at 4 °C under refrigeration. Every three days, 5 mg was taken out and hydrolyzed by adding 4 mL of substrate(p-NPL) at a concentration of 0.3 mg/mL under its optimal conditions (pH 9, temperature 50 °C), while three parallel controls were made, and the storage stability was derived after the completion of the reaction by calculating the relative activities of the immobilized lipase at different storage times.

### 2.5. Enzyme Load Testing

**Figure 2:**
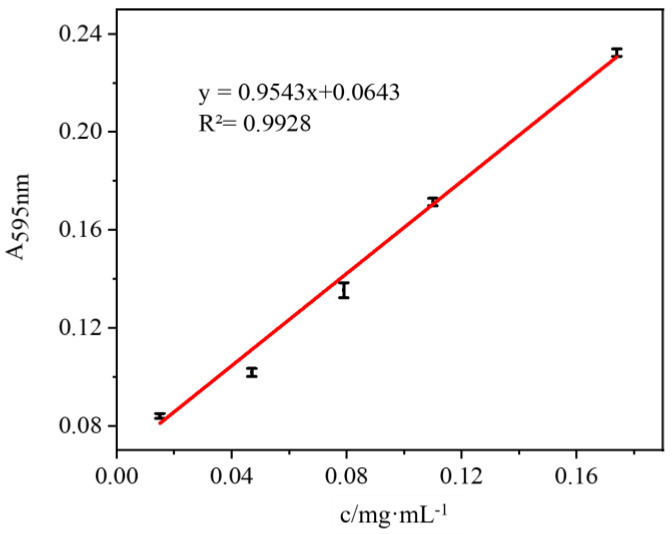
Standard curve of enzyme concentration.

Bradford assay was used to determine the enzyme load. At the end of the immobilization process, the immobilized system was put into ultrafiltration centrifuge tubes to remove the unfixed enzyme. After that, the absorbance was measured at 595 nm, and the standard curve was plotted as in Figure 2. The absorbance of the test solution was measured at 595 nm. Then, the protein concentration of the unknown liquid was obtained by substituting the measured absorbance into Equation (1). Finally, the enzyme loading P was calculated based on the initial amount of enzyme. The immobilized amount was 418 μg/mg, which resulted in an immobilization rate of 79.9%.

(1) $$P=\frac{\left(c_{0}v_{0}-c_{1}v_{1}\right)}{s}$$

$c_{0}$: concentration of enzyme solution before fixation (mg/mL);

$c_{1}$: concentration of enzyme in the solution to be measured (mg/mL);

$v_{0}$: volume of enzyme solution added to the carrier during fixation (mL);

$v_{1}$: volume of liquid to be tested (mL);

*s*: area of carrier at fixation (cm^2^).

### 2.6. Study on the Environmental Performance of Immobilized Lipase

(1) The catalytic activity test of lipase was conducted according to reference [31].
(2) Biological risk testing of soil degradates

The luminescence intensity of Vibrio fischeri was used to characterize the biological risk of biodegradants. A total of 20 g of blank leather shavings and leather shavings after immobilization of PPL were placed in soil-filled flower pots. Then, 1 g of the blank leather shavings and the degraded soil of the leather shavings after immobilizing PPL was dispersed in 40 mL of pure water for 48 h and then centrifuged to take the supernatant, which was diluted 10-fold with 2 wt % NaC1 solution; 2 wt % NaCl was used as a blank control, and 10 mg/L zinc sulfate heptahydrate was used as a positive quality control. Then, 180 μL of test solution and 20 μL of bacterial solution were added to the microtiter plate. The bioluminescence intensity was tested at 0 min and 20 min using a multifunctional enzyme marker [32].

(3) Hydrolysis efficiency test of immobilized enzymes

Castor oil was used to simulate the waste oil produced by tanning. A total of 12 g of castor oil was dissolved in 35 mL of deionized water, and then 0.87 mg of immobilized PPL and an equal amount of free PPL were added, respectively. After the reaction was carried out for 20 min, 40 min, 60 min, 80 min, and 100 min, the upper 2 g of oil was extracted to detect the acid value of castor oil, to determine the catalytic hydrolysis efficiency of free lipase and immobilized PPL on castor oil [33]. The formula for the acid value is shown in Equation (2).

(2) $$V=\frac{V_{\max}+\left[S\right]}{K_{m}+\left[S\right]}$$

*V*: Reaction rate (mmol/L/min);

*V_max_*: Maximum reaction rate (mmol/L/min);

[*S*]: Concentration of substrate (mg/mL);

*K_m_*: Substrate concentration at maximum reaction rate.

(4) Determination of optimal catalytic conditions

A total of 5 mg of PPL-immobilized leather shavings and the same amount of free PPL (2.4 mL) were placed in a test tube. Then, 4 mL of substrate was added with a concentration of 0.3 mg/mL at a temperature of 40 °C. The reaction was carried out in PBS buffer at pH values of 6, 7, 8, 9, 10, and 11, respectively. The optimum pH of the free enzyme and immobilized enzyme reaction was determined by calculating the relative activity at different pH values. Similarly, the same amount of free enzyme and immobilized enzyme was placed in PBS buffer at the optimal pH, and the enzyme activity was measured at temperatures of 30 °C, 40 °C, 50 °C, 60 °C, and 70 °C, respectively. At the same time, three groups of parallel controls were conducted. After the reaction was completed, the optimum temperature of the reaction between free enzyme and immobilized enzyme was determined by calculating the relative activity at different temperatures [34].

## 3. Results and Discussion

### 3.1. Ultra-Depth-of-Field Microscope Analysis

**Figure 3:**
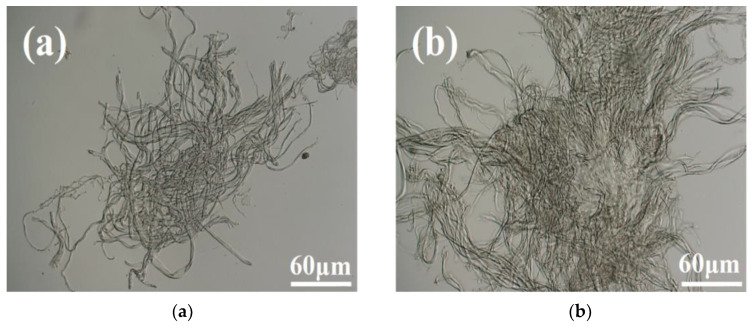
The ultra-depth-of-field microscopy images (**a**) Blank leather shavings. (**b**) Leather shavings with immobilized PPL.

Figure 3 shows the ultra-depth-of-field microscopy images of blank leather shavings (a) and leather shavings with immobilized PPL (b). As can be seen in Figure 3a, the gaps between the blank leather shaving carriers are large and dispersed. Compared with the blank leather chip carriers, the fiber of the leather chip after enzyme immobilization shows obvious changes, and the particle size of the leather chip becomes larger and denser due to the coating of hydrogel, which preliminarily proves that the enzyme system embedded in the hydrogel network had been formed.

### 3.2. SEM Analysis

**Figure 4:**
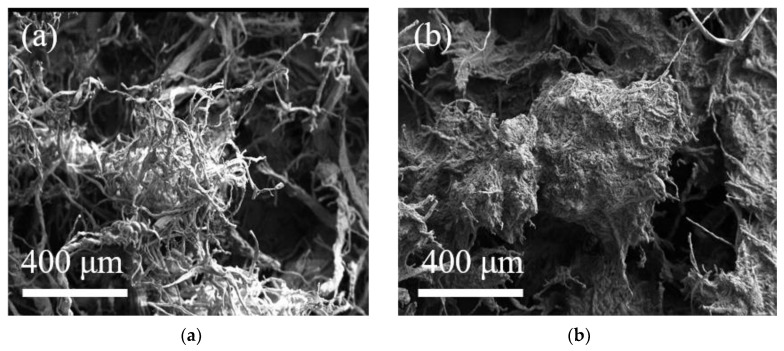
SEM (**a**) Blank leather shavings. (**b**) Leather shavings with immobilized PPL.

The results of ultra-depth-of-field microscopy showed that the surface of leather microspheres was successfully grafted with a hydrogel-embedded enzyme system. In order to further observe the changes in the surface morphology of the leather shavings before and after grafting, the blank leather shavings and the leather chips with immobilized PPL were characterized by SEM. It can be clearly observed from Figure 4a that the gaps between the blank leather shavings carrier are large and loosely distributed. Compared with the blank leather chip carrier, it can be found that the pores between the leather shaving fibers were filled after immobilization, and the hydrogel can be clearly observed, which further verifies the formation of the enzyme system embedded in the hydrogel network.

### 3.3. XPS Analysis

**Figure 5:**
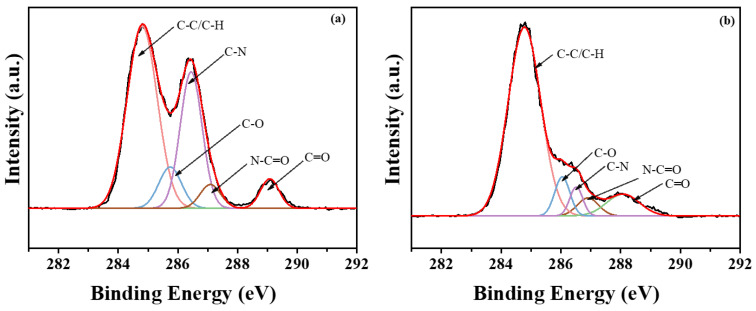
C1s spectra of blank leather shavings (**a**) and leather shavings with immobilized PPL (**b**).

The energy spectrum of C1s on the surface of blank leather shavings and leather shavings with immobilized PPL was analyzed by XPS. In Figure 5, the C1s spectra of blank leather shavings and PPL immobilized on leather shavings can be divided into five peaks, C-H and C-C (285.0 eV), C-O peak (286.6 eV), C=O peak (288.6 eV), N-C=O peak (287.2 eV), and C-N peak (285.9 eV). Compared with the content of chemical bonds in the C1s energy spectrum of Figure 4a (0.53:0.28:0.09:0.05:0.05), the content of chemical bonds in the C1s energy spectrum of the immobilized lipase of leather shavings in Figure 4b (0.75:0.13:0.06:0.06:0.003) was significantly changed, which proves the successful grafting of the hydrogel-embedded enzyme system.

### 3.4. FTIR Analysis

**Figure 6:**
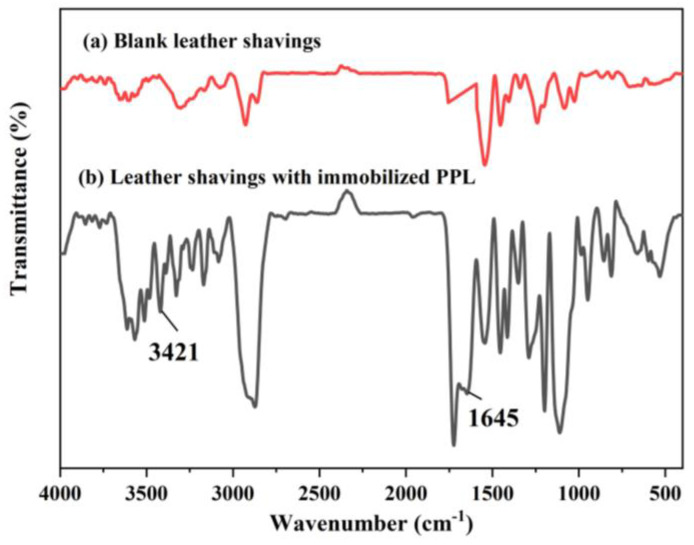
Infrared spectra of blank leather shavings (**a**) and leather shavings with immobilized PPL (**b**).

To determine the changes in characteristic groups on the surface of leather chips before and after visible light grafting polymerization, FT-IR tests were performed. Figure 6 shows the infrared spectra of blank leather shavings (Figure 6a) and leather shavings with immobilized PPL (Figure 6b), respectively. Compared with Figure 6a, Figure 6b shows obvious absorption peaks at 1645 cm^−1^ and 3421 cm^−1^. This is mainly caused by the C=O stretching vibration in diaryl ketones and the C-H stretching vibration on the benzene ring, which further explains the formation of the hydrogel-embedded enzyme system.

### 3.5. Catalytic Properties of Immobilized Lipase

#### 3.5.1. The Optimal Catalytic pH for Immobilized Lipase

**Figure 7:**
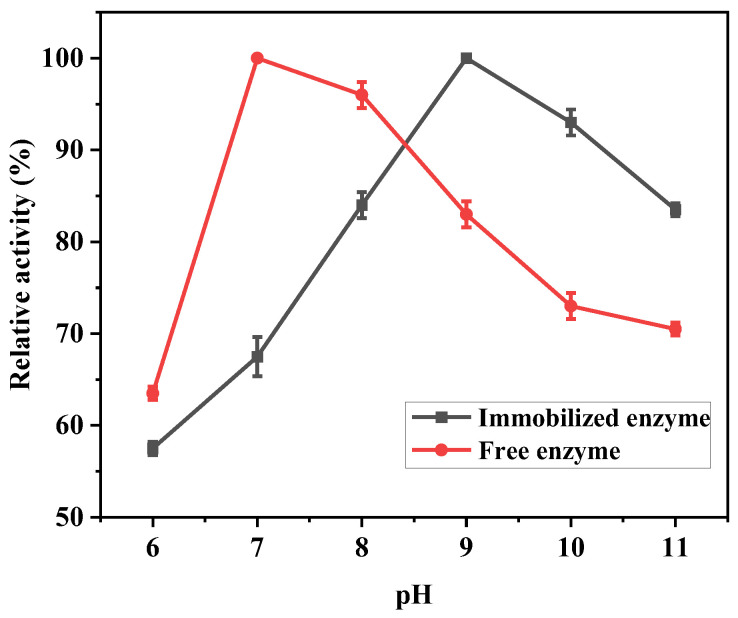
The effect of solution pH on the catalytic activity of free and immobilized enzymes at a temperature of 40 °C.

There are many factors that can affect the catalytic activity of lipase, and one important factor is pH. The charged state of the enzyme will change with the change in pH, which will affect the dissociation state of the enzyme protein, and then change the structure of the catalytic site of the enzyme, and finally affect the catalytic activity of the enzyme. In order to investigate the effect of pH on the hydrolysis of free and immobilized enzymes, the changes in catalytic activities of free and immobilized enzymes were measured at 40 °C and different pH. As shown in Figure 7, the relative activities of free enzyme and immobilized enzyme showed a tendency of decreasing in the early stage and recovering in the late stage with the increase in pH value. In the range of pH 6–11, the activity of the immobilized enzyme fluctuated by 15%, which was significantly lower than that of the free enzyme (30%), indicating that the immobilized enzyme has a wider adaptive range to pH change. The immobilized enzyme maintained 85% activity at pH 11, which was 6.25% higher than that of the free enzyme, further demonstrating its stability under extreme pH conditions. This enhanced stability is mainly attributed to the embedding of the immobilized enzyme into the 3D network structure of the hydrogel, which results in a significant reduction in the disruption of the enzyme’s active center upon changes in the catalytic environment. As a result, the immobilized enzyme has superior catalytic performance.

#### 3.5.2. Optimum Catalytic Temperature

**Figure 8:**
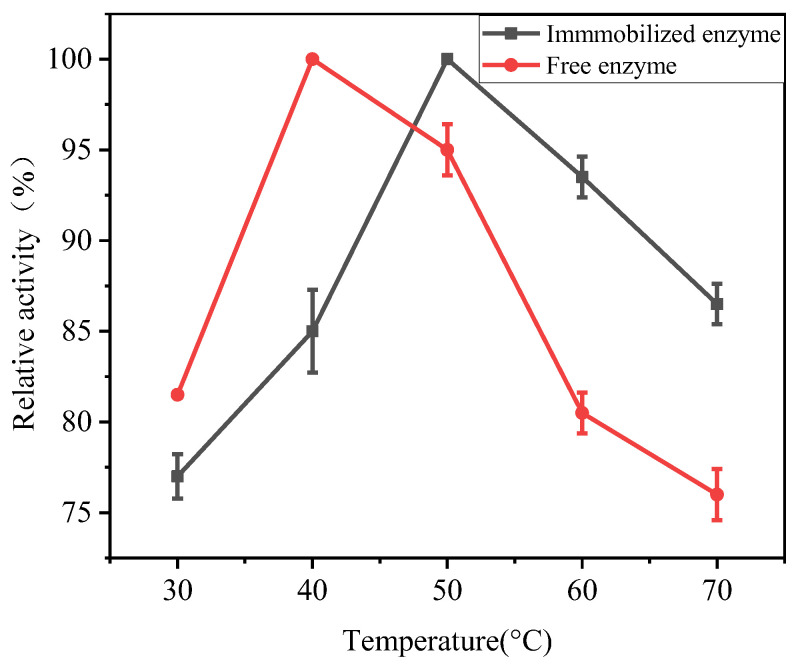
The effect of reaction temperature on the catalytic activity of free and immobilized enzymes (pH = 9).

Temperature is another important causative factor affecting enzyme activity. In order to investigate the effect of temperature on the activities of free and immobilized enzymes, the relative activities of free and immobilized enzymes were determined by placing them in PBS buffer with their respective optimal pH values under a series of temperature gradients. As shown in Figure 8, with the increase in temperature, the relative activity of enzymes first increased and then decreased. The relative activity of the free enzyme reached the highest at about 40 °C, and the relative activity of the immobilized enzyme reached the highest at about 50 °C. This is mainly due to the significant difference between the actual temperature and the enzyme’s optimal temperature, which can affect the enzyme’s protein structure and lead to enzyme inactivation [35]. When the temperature is low, whether the free enzyme or immobilized enzyme, the reaction activity and catalytic ability do not reach the highest value. As the temperature gradually increases and approaches the optimal temperature, the enzyme activity also increases. When the temperature is higher than the optimal temperature, the structural stability of the enzyme itself is affected and gradually deactivated. Therefore, in the latter half of the curve, the relative activity of the enzyme gradually decreases with the increase in temperature. However, the relative deactivation rate of the immobilized enzyme is slower than that of the free enzyme. This indicates that the immobilized enzyme has better high-temperature resistance. This is mainly due to the protective effect of the three-dimensional network structure of hydrogel on the conformation of enzymes [36].

#### 3.5.3. Determination of Storage Stability

**Figure 9:**
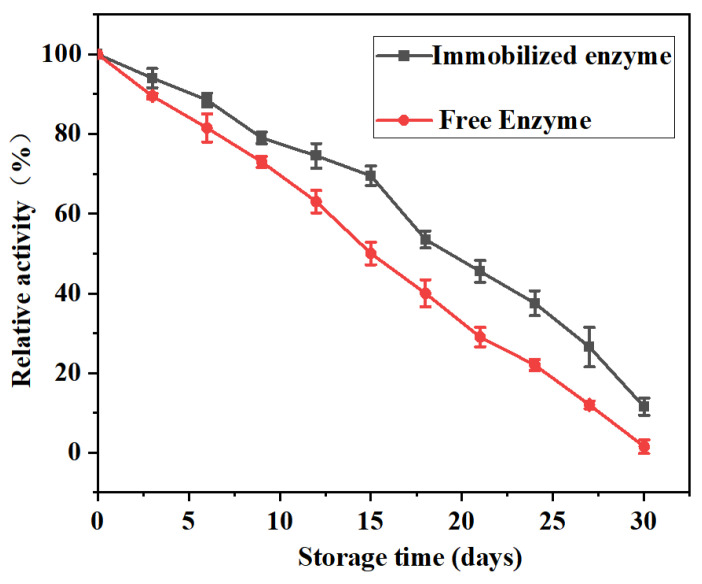
Storage stability curves of free and immobilized enzymes.

The storage stability of enzymes is one of the constraints in the commercial application of enzymes. The easy decomposition of enzymes and the susceptibility of their activity to external environmental influences can make long-term storage and long-distance transport of enzymes very difficult. This feature is not conducive to the centralized and large-scale preparation of industrial enzymes. When the storage stability of the enzyme is higher, it is also more conducive to the recycling of the enzyme, thus reducing the cost of enzyme industrial application. In this experiment, the storage stability of free and immobilized enzymes was tested. As shown in Figure 9, the relative activities of free and immobilized enzymes decreased substantially with the increase in storage time. However, the loss of free enzyme activity was faster and almost completely inactivated after 30 days. Compared with the free enzyme, the immobilized enzyme still retained about 15% of its original activity after 30 days, and the deactivation rate was significantly slower. It can be seen that the storage stability of lipase has been significantly improved after immobilization.

#### 3.5.4. Hydrolysis Efficiency of Immobilized Enzymes

**Figure 10:**
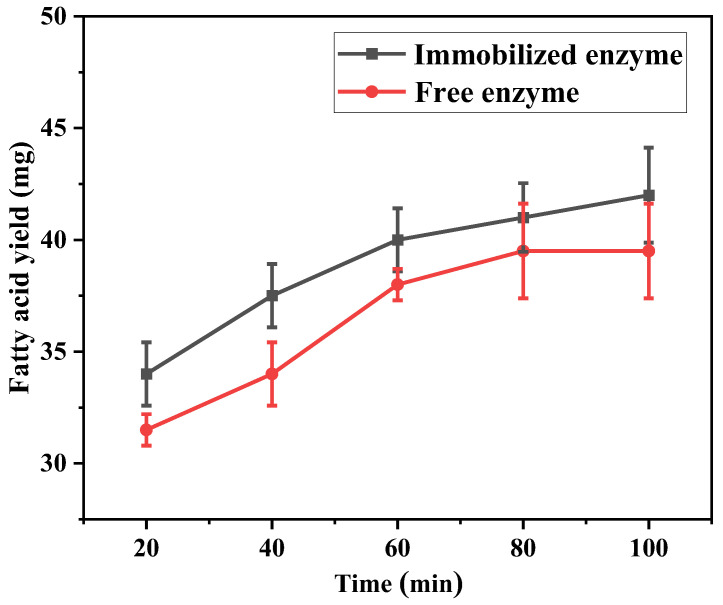
Hydrolysis yield curves of immobilized PPL and free PPL of leather shavings.

To explore the feasibility of this system in actual waste oil treatment and to contribute to the realization of waste resources, the hydrolysis efficiency of the immobilized enzyme on castor oil was measured. As can be seen from Figure 10, with the increase in time, the hydrolysis efficiency of the free enzyme and immobilized enzyme gradually increased and finally stabilized, and the hydrolysis yield of the immobilized enzyme was greater than that of the free enzyme. The hydrolysis efficiency of the free enzyme was 39.5 μg/min and that of the immobilized enzyme was 42 μg/min at 100 min of reaction. Due to that, the immobilized enzyme has a more stable structure after the visible light polymerization technology, which can maintain the spatial conformation of the enzyme in the reaction. The catalytic active site has more frequent contact with the reaction substrate and thus has higher catalytic activity.

#### 3.5.5. Soil Toxicity Testing of Immobilized Enzymes

**Figure 11:**
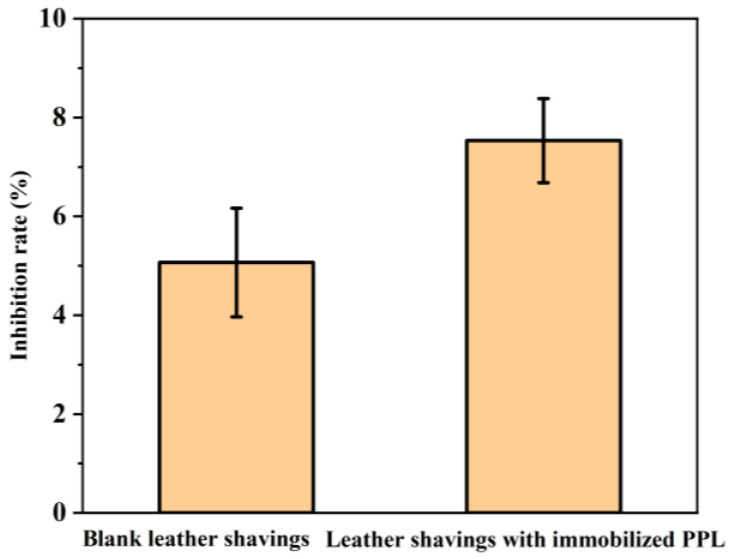
Inhibition of bioluminescence of soil extracts by blank leather shavings and leather shavings with immobilized lipase.

Hazardous chemicals in carriers may contaminate soil. Therefore, soil toxicity of degraded materials is also an important concern. The biological risk of soil extracts from the degradation of blank leather chips and leather-chip-immobilized PPL was evaluated. Bioluminescence inhibition of Vibrio fischeri, an important indicator of toxicity, was determined by diluting the soil degradation product extracts containing blank leather and leather-immobilized PPL the same number of times and assaying them. As shown in Figure 11, the bioluminescence inhibition rate of blank leather shavings’ degradation products in soil extracts is 5.07%, and that of leather shavings with immobilized lipase in soil extracts is 7.50%. The results showed that both blank leather shavings and leather shavings with immobilized PPL have low biological risks. The good biodegradability and low biological risk enable immobilized enzymes to have excellent environmental compatibility, and they will not cause secondary pollution to the environment in the treatment of waste oil.

#### 3.5.6. Circulating Stability Testing of Immobilized Lipase

**Figure 12:**
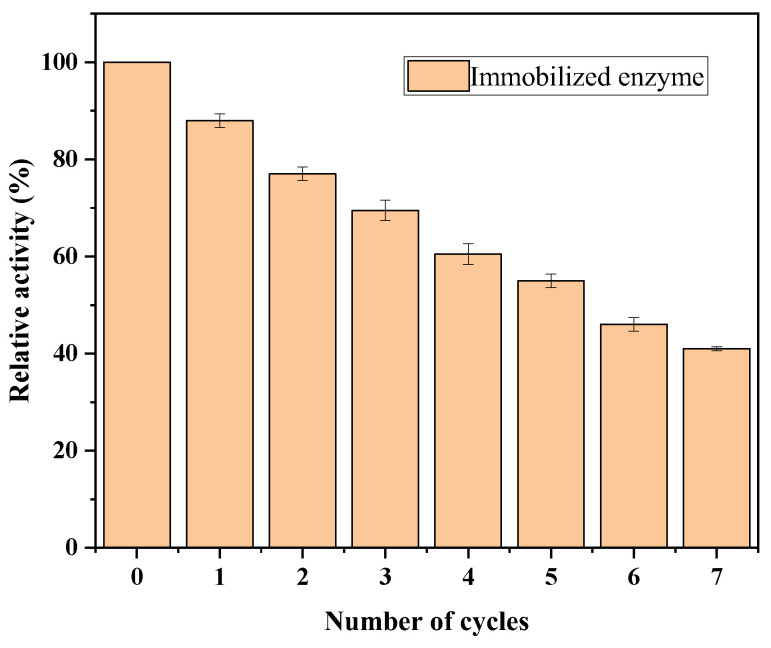
Operational stability of the immobilized enzyme at a temperature of 50 °C and pH = 9.

As shown in Figure 12, the relative activity of the immobilized enzyme showed a steady decline with the increase in the number of cycles. However, it is worth noting that even after seven cycles, the relative activity of the immobilized enzyme can still be maintained at around 40%, indicating that the catalytic activity of the immobilized enzyme still has a high level after multiple repeated use. This is mainly due to the stable encapsulation effect of the hydrogel network structure on the enzyme, which can prevent the leakage of the enzyme and achieve a high recycling rate.

## 4. Conclusions

In this paper, lipase was fixed on the surface of organic chromium-free tanned leather chips by visible light-controlled active grafting polymerization. Subsequently, the structure and properties of the immobilized lipase system were characterized. The results showed that the hydrogel-embedded enzyme system was formed successfully. The optimal pH of the immobilized enzyme was about 9, and the optimal temperature was 50 °C, which had a wide adaptive range. The relative activity of the immobilized enzyme could be maintained at about 40% after seven cycles, and it was inactivated after 30 days of storage, which improved the cycling and storage stability of the immobilized enzyme. The cycling stability and storage stability of the immobilized enzyme were improved compared with those of the free enzyme. The results of hydrolysis experiments showed that the immobilized enzyme has better catalytic activity and hydrolysis efficiency than the free enzyme. In the treatment of waste oil, the immobilized system has good biocompatibility, and will not pollute the environment, so as to achieve “treating waste with waste”, and to improve the added value of the tanning solid waste of the organic chrome-free tanning system.

## Data Availability

The original contributions presented in this study are included in the article. Further inquiries can be directed to the corresponding authors.
